# Asian elephant interferons alpha and beta and their anti-herpes viral activity

**DOI:** 10.3389/fimmu.2025.1533038

**Published:** 2025-03-25

**Authors:** Jonathan Haycock, Tanja Maehr, Akbar Dastjerdi, Falko Steinbach

**Affiliations:** ^1^ Faculty of Health and Medical Sciences, University of Surrey, Guildford, United Kingdom; ^2^ Department of Virology, Animal and Plant Health Agency, Addlestone, United Kingdom

**Keywords:** EEHV, elephant, innate immunity, interferon, herpesvirus

## Abstract

The type I interferons (IFNs) are a group of key cytokines of the vertebrate innate immune system that induce an antiviral state in uninfected cells. Experimental *in-vitro* and *in-vivo* data have proven the fundamental role these cytokines possess in the protective response to a wide variety of pathogens, including herpesviruses. In a clinical setting, IFNs have been an important treatment in humans for several decades and increasing evidence demonstrates their potential in controlling viral haemorrhagic fevers when administered early in disease. In juvenile Asian elephants, elephant endotheliotropic herpesvirus haemorrhagic disease (EEHV-HD) often proves fatal when an effective adaptive immune response cannot be mounted in time, suggesting that an enhancement of the innate immune response could provide protection. This study sequenced six members of the Asian elephant type I IFNs, most closely related to sequences from the African elephant and Florida manatee. Subsequently, recombinant Asian elephant IFNα and IFNβ proteins were expressed and assessed for bioactivity *in-vitro*, relative to recombinant human IFNs, using a novel infection model incorporating primary Asian elephant fibroblasts and bovine alphaherpesvirus 1 (BoHV-1) as a surrogate for EEHV. In a dose-dependent manner, both Asian elephant IFNs and human IFNα2a protected cells from BoHV-1 infection in this proof-of-concept study, even if applied up to 24 hours post-infection *in-vitro*.

## 1 Introduction

Interferons (IFNs) are a conserved family of pleiotropic cytokines, identified across all vertebrate species (1, 2). Type I IFNs (IFN-I) have been identified through their antiviral effects, but alongside their immunomodulatory activities (3–5), they provide a crucial stimulatory bridge between the innate and adaptive immune responses (6–10). IFNα is a multigene cluster in mammals, whereas only one IFNβ gene exists in humans, mice and horses (11, 12). Once IFN-I bind to their receptor, IFN signalling via the JAK/STAT pathway results in the transcription of hundreds of interferon-stimulated genes, [ISGs; (13–16)], proven to be vital in the immune response to invading pathogens, including herpesviruses (17, 18).

Elephant endotheliotropic herpesviruses (EEHVs), also known as Elephantid betaherpesviruses, are a group of potentially fatal viruses in the genus *Proboscivirus*, within the *Betaherpesvirinae* (19, 20). Since the initial cases, over 100 fatalities associated with EEHV haemorrhagic disease (EEHV-HD) have been reported in both *ex-situ* (captive) and *in-situ* (both wild and captive in range countries) juvenile Asian elephants (*Elephas maximus*) between the ages of 1 and 8 years old (21–27). Since their initial identification in 1995 (28), the EEHV-HD mortality rate in captive Asian elephants has been reported to be as high as 90% (29). Additionally, as no anti-viral compound has yet proven effective against EEHVs, these viruses remain among the highest threats to the sustainability of captive populations of this endangered species (30–34).

Although the clinical disease associated with EEHVs is often acute, clinical signs are preceded by a period of subclinical infection (35–37) before an exponential increase in blood EEHV load and disease manifestation (38, 39). Therefore, the application of veterinary antiviral compounds at a sufficiently early stage of infection may prove beneficial in limiting the development of EEHV-HD.

The exogenous administration of IFNs has been widely applied to viral diseases of humans, establishing them as important therapeutics, most notably in relation to hepatitis B and C (40–45). Additionally, IFNs have emerged as a crucial part of the innate response to viral haemorrhagic fevers, with an early and sustained response correlated with survival (46–50). Therefore, the clinical application of IFN-I against EEHV infections warrants further investigation. However, the lack of elephant IFN-I interferons, and the ongoing inability to culture EEHVs *in-vitro*, has hindered more targeted research.

This study aimed to characterise Asian elephant type I IFNs, including their gene sequences, expression of the associated proteins, and evaluation of their *in-vitro* antiviral bioactivity, relative to human IFNs, using an *in-vitro* herpesviral infection model. The preliminary results of this study may provide additional interventional strategies to boost the Asian elephant innate immune system in the early stages of EEHV infections to prevent progression to clinical disease.

## 2 Materials and methods

### 2.1 Animals and sample collection

The single Asian elephant used in this study was a seven-year-old captive-born female housed at a zoological collection within the United Kingdom. The animal was clinically healthy at the time of sample collection. Blood samples were collected under trained behaviour into ethylenediaminetetraacetic acid (EDTA) blood tubes for the purpose of routine EEHV monitoring and/or diagnostics according to routine veterinary practices. Veterinary interventions such as blood sample collection for the purpose of clinical health screening fall under the Veterinary Surgeons Act 1966 and do not require further ethical approval, as they are intended solely to maintain animal welfare. Samples were sent at ambient temperature overnight to the laboratory and refrigerated at 4-8°C until being processed within 2 hours.

### 2.2 Extraction of genomic DNA (gDNA)

Extraction of gDNA from 140 µL aliquots of whole EDTA blood was performed using the QIAamp Viral RNA Mini Kit (Qiagen, Manchester, UK) according to the manufacturer’s instructions and samples were eluted into a final volume of 60 μL of buffer AVE.

### 2.3 Identification of Asian elephant IFN genes

The published sequences from six Asian elephant genomes [NCBI SRA database https://www.ncbi.nlm.nih.gov/sra; accession numbers SRS927124, SRS927123, SRS927126, SRP065915, SRS1158889, ERS365881 and ERS365882; (51, 52)] were used in combination with the online NCBI BLAST tool (https://blast.ncbi.nlm.nih.gov/Blast.cgi) for the assembly of predicted Asian elephant IFNα and IFNβ genes. The published corresponding gene sequences of the horse (*Equus caballus*; accession numbers NM_001099441, NM_001099440), and predicted corresponding gene sequences of the African elephant (*Loxodonta africana*; accession numbers XM_003407658.2, XM_003407655.2, XM_023545333.1, XM_003407652.2, XM_003407651.2, XM_003407333.2, XM_023545332.1, XM_010587902.2, XM_003407336.2) were used as respective templates in predicting IFNs of Asian elephants. The 5’ and 3’ untranslated regions (UTRs) of the IFN genes were also included in their assemblies. Primers were designed within the UTRs either side of the 5’ and the 3’ ends of the predicted IFN genes to verify entire coding sequences through polymerase chain reaction (PCR) and Sanger sequencing.

Primers used in this study were synthesised by Eurofins Genomics (Ebersburg, Germany; **Table 1**). PCRs were performed in a Veriti Thermal Cycler (Life Technologies, Paisley, UK) and, as target genes were predicted to be intronless, gDNA was used as a template. Each PCR contained 10 μl of Fast Cycling PCR Master Mix (Qiagen), 2 pmol each of forward and reverse primer, 2 μl of gDNA and nuclease-free water to a final reaction volume of 20 μl. Thermocycling was performed for five minutes at 95°C, followed by 40 cycles of 95°C for 15 seconds, 55°C for 30 seconds and 72°C for one minute.

**Table 1:** Primers used in this study to sequence Asian elephant IFN genes.

Amplicon	Forward primer	Reverse primer
IFNα1	GCCCAGAGCAAGGTCTTCRGAGAAC	AGAAATGMGAGTCTTTGA
IFNα2	AGCAAGGTCTTCAGAGAACCTGGAG	TTRTAGCAGAAATGAGAGYCTT
IFNα3	CTTCRGARAACCTGGAGGCCCAGGC	GAGYRTATTAGTYRATGASAATC
IFNα4	AGAACCTGGAGGCCCAGGCTCACAG	GAGTCTTTGAAATGGCTRAA
IFNαPG	GCCTCACCGCCATCAACC	CCTGATTGTAGCAGAAATGAGAGTC
IFNβ	GCCCATACCCATGGAGGAAAG	TGTCAGCACGGTCCGGTCC
T7	TAATACGACTCACTATAGGG	CTAGTTATTGCTCAGCGGTG

A set of primer pairs were designed for amplification of IFNα family genes of Asian elephants, and one primer pair was designed for the amplification of the single predicted IFNβ gene. Sequencing primers spanning the multiple cloning site of pET303CT-His vectors are also shown (T7).

Aliquots of each PCR product were mixed at a ratio of 5:1 with 6x Blue/Orange Loading Dye (Promega UK Ltd.), separated through electrophoresis in a 2% agarose gel containing SYBR^®^ Safe DNA Gel stain (Life Technologies) and visualised under ultraviolet light using a Universal Hood II transilluminator (Bio-Rad, Watford, UK). DNA bands of the predicted size were cut from the gel and purified using the MinElute^®^ Gel Extraction Kit (Qiagen) according to the manufacturer’s instructions.

Purified DNA amplicons were subjected to a single run of Sanger sequencing at the APHA Central Sequencing Unit. The nucleotide (NT) and deduced amino acid (AA) sequences were analysed via NCBI BLAST and the Protein Homology/Analogy Recognition Engine V 2.0 [Phyre^2^; http://www.sbg.bio.ic.ac.uk/phyre2/html/page.cgi?id=index; (53)] respectively. All NT and AA sequence alignments were performed using ClustalW in MegAlign 15.0 (DNASTAR).

### 2.4 Cloning Asian elephant IFN genes

Amino acid sequences of the IFNα (EleIFNα1) and IFNβ (EleIFNβ) genes were analysed for signalling peptides using the SignalP v 4.0 online tool [http://www.cbs.dtu.dk/services/SignalP/index.php; (54)]. The signalling peptide NT sequences and the stop codon were then excluded from each gene sequence. The IFNα and IFNβ sequences were each codon-optimised for expression in *Escherichia coli* (*E.coli*) using Eurofins Genomics online software (https://www.eurofinsgenomics.eu/en/home), synthesised, and sub-cloned into Invitrogen™ Champion™ pET303CT-His plasmid vectors (Fischer Scientific, Loughborough, UK).

### 2.5 Bacterial expression and purification of IFN proteins

The pET303CT-His plasmid vectors were transformed into BL21(DE3) Competent Cells (New England Biolabs, Hitchin, UK) according to the manufacturer’s instructions. Up to 100 µL of each transformation reaction was spread over LB agar plates infused with 100 μg/mL ampicillin, and single colonies were picked the following day to inoculate a 10 mL starter culture of selective LB broth (containing 100 μg/mL ampicillin).

Starter cultures were screened after 24 hours for gene inserts using T7 sequencing primers (**Table 1**) in a PCR as described above but only running for 35 cycles. Amplicons were verified by visualisation of the predicted size DNA band following agarose gel electrophoresis. Aliquots of each starter culture were then added to an expression culture of selective LB broth. Expression cultures were incubated at 37°C and approximately 250 rpm until the optical density, at a wavelength of 600 nm, reached 0.5 to 0.8. Protein expression was then induced with isopropyl β-D-1-thiogalactopyranoside (IPTG) at a final concentration of 400 µM for between three and five hours at 37°C. Bacteria were harvested by centrifugation at 5,000 x *g* for 15 minutes at 4°C, the supernatant was discarded, and individual cell pellets were weighed before storage at -20°C.

Bacterial cell pellets were thawed at room temperature before 10 mL B-PER™ Complete Bacterial Protein Extraction Reagent and 100 μL 10x Halt™ Protease Inhibitor Cocktail (both Fisher Scientific) were added per gram of cell pellet. Cell pellets were resuspended by vortexing and incubated for 20 minutes at room temperature in a bench-top shaker at 150 rpm.

Solubilisation of bacterial inclusion bodies was performed using a combination of sodium lauroyl sarcosinate (sarkosyl), Triton X-100 and 3-[(3-cholamidopropyl)dimethylammonio]-1-propanesulfonate (CHAPS) detergents similar to previous reports (55, 56). Sarkosyl (Sigma Aldrich, Gillingham, UK) was added to a final concentration of 2% and incubated at room temperature in a bench-top shaker at 150 rpm for at least two hours. This was followed by centrifugation at 20,000 x *g* for 20 minutes at 4°C and the supernatant was transferred to a new tube and stored. The process was repeated with the insoluble pellet but sarkosyl was added to a final concentration of 10%. The supernatants were combined, and Triton X-100 and CHAPS (both Sigma Aldrich) were added to final concentrations of 4% and 40 mM, respectively.

Purification of the His-tagged Asian elephant IFN proteins was performed using 1 mL tubes of the Thermo Scientific™ HisPur™ Ni-NTA Purification Kit (Fisher Scientific) according to the manufacturer’s protocol. Purified protein preparations were concentrated using Amicon^®^ Ultra-15 10K Centrifugal Filter Devices (Millipore Corporation, Watford, UK) at 4,000 x *g* for 20 minutes.

Residual imidazole was removed from the soluble protein preparations using Zeba™ Spin Desalting Columns (5 mL size) (Fisher Scientific) according to the manufacturer’s instructions. Final protein preparations in sterile PBS were divided into 50 µL aliquots and stored until further use at -80°C.

Dilutions of these protein preparations were made in sterile PBS and quantified using the Thermo Scientific™ Coomassie Plus (Bradford) Assay Kit (Fisher Scientific) according to the manufacturer’s “microplate procedures” protocol. All standards and samples were measured in triplicate. The absorbance of each sample at 595 nm was measured using an Infinite^®^ M Plex plate reader (Tecan UK Ltd., Reading, UK).

### 2.6 SDS-PAGE and Western blot

Aliquots of protein preparations were added to 5 µL of 4x Bolt™ Lithium Dodecyl Sulphate (LDS) Sample Buffer, 2 µL of 10x Bolt ™ Reducing Agent and sterile deionised water to a final volume of 20 µL. The samples were heated for 10 minutes at 70°C and loaded onto a Bis-Tris Plus 4-12% gradient polyacrylamide gel in 2-(N-morpholino) ethanesulfonic acid (MES) SDS buffer containing antioxidant (all Fisher Scientific). Invitrogen™ iBright™ Prestained Protein Ladder (11-250 kDa; Fisher Scientific) and BLUeye™ Prestained Protein Ladder (10-245 kDa; Geneflow Ltd., Lichfield, UK) were also loaded onto the gel to monitor protein separation. Alternatively, SeeBlue^®^ Pre-Stained Protein Standard (3-198 kDa; Fisher Scientific) replaced the BLUeye™ Protein Ladder. Electrophoresis was carried out at 200 volts for 25 minutes.

For Coomassie staining, gels were washed in sterile deionised water and immersed in Coomassie Brilliant Blue R-250 staining solution (Bio-Rad) for one hour. The staining solution was replaced with de-staining solution (40% methanol, 10% glacial acetic acid, 50% distilled water) until background staining had been removed and the bands of the protein ladder and/or sample proteins were clearly visible. The gels were visualised using an Azure c280 imaging system (Cambridge Bioscience, Cambridge, UK).

For Western blots, protein bands were transferred to nitrocellulose membranes using the Invitrogen™ iBlot™ 2 Dry blotting system (Fisher Scientific) according to the manufacturer’s instructions. The membrane was then washed in sterile deionised water and immersed in 5 mL of 1x iBind™ solution (Fisher Scientific). Membrane blocking and antibody binding were performed using the iBind™ Western System (Fisher Scientific) according to the manufacturer’s instructions. A primary monoclonal mouse anti-His tag antibody (Sigma Aldrich; Clone 6AT18) was diluted 1:1,000 and the secondary polyclonal rabbit anti-mouse IgG – horseradish peroxidase antibody (Sigma Aldrich) was diluted 1:16,000. After incubations, the membrane was washed twice in 20 mL of sterile distilled water for two minutes and then immersed in 7.5 mL of Thermo Scientific™ SuperSignal™ West Pico PLUS Chemiluminescent Substrate working solution (Fisher Scientific) at room temperature for five minutes. The membrane was placed in a clear plastic wrap and imaged using an Azure c280 imaging system.

### 2.7 Primary Asian elephant fibroblast cell culture

Primary Asian elephant preputial fibroblast (EF) cells were cultured in a 75 cm^2^ cell culture flask containing EF medium consisting of: Gibco™ Minimum essential medium (Fisher Scientific), 10% FBS, Penicillin-Streptomycin (10,000 units penicillin/mL, 10,000 µg streptomycin/mL) and Nystatin (10,000 units/mL; Fisher Scientific). Upon reaching 75 to 80% confluency, cells were passaged or seeded into 24-well plates at 50% confluency per well.

### 2.8 Herpesviral infection of elephant fibroblasts

As attempts to culture EEHVs have so far been unsuccessful (57–59), infections of primary Asian elephant fibroblasts were trialled with two other herpesviruses, to ascertain whether either was capable of inducing a measurable cytopathic effect (CPE) in this cell type. Serial tenfold dilutions (10^-1^ to 10^-8^) were made in Dulbecco’s Modified Eagle Medium (DMEM) of BoHV-1 Oxford strain with a titre of 10^7.5^ PfU/mL and Equine alphaherpesvirus 1 (EHV-1) strain V592. These viruses were chosen in part for their availability during this proof-of-concept study, but also based on practical considerations during infection assays, compared to the phylogenetically more closely related human cytomegalovirus (HCMV) or human herpesvirus 6 (HHV-6). A 100 µL aliquot of each virus preparation or 100 µL of DMEM for no-virus-controls was added to each well. Plates were sealed and incubated at 37°C and 5% CO_2_ for one hour. Each well was subsequently washed three times with 1.5 mL DMEM medium before 1.5 mL EF medium was applied, the plate was re-sealed, and incubated for up to seven days.

EF cells were fixed with neutral buffered 10% formalin (Sigma Aldrich) for at least 40 minutes. Formalin was then replaced with 250 µL of 2.3% Crystal Violet solution (Sigma Aldrich). Plates were incubated at room temperature for five minutes, washed and air-dried. Assessment of cell monolayers was performed using a Leica DM IL LED microscope (Leica Microsystems UK Ltd., Milton Keynes, UK). Over the course of the study, BoHV-1 infection of Asian elephant fibroblasts (without the addition of IFNs) was investigated in five experimental replicates.

### 2.9 Effect of IFN on herpesviral infections of elephant fibroblasts

Initial investigations of the bioactivity of human and Asian elephant IFNs were carried out using 1:100 dilutions of unquantified Asian elephant recombinant IFNα1 and β preparations (rEleIFNα1, rEleIFNβ) alongside a control preparation from the insert-less pET303CT-His plasmid vector. Similarly, dilutions were made of recombinant human interferon alpha 2a (hIFNα2a) and human interferon beta 1a (hIFNβ1a; both Immunotools, Friesoythe, Germany) to produce preparations of 1,000 international units (IU)/mL. All preparations were produced in DMEM medium.

In three experimental replicates, EF medium was removed from each well and 1 mL aliquots of unquantified IFN solutions were added 8 hours prior to infection with BoHV-1.

For a subsequent investigation of the time point at which IFNs no longer protect EF cells from BoHV-1 infection, serial 1:10 dilutions were made of quantified rEleIFNα1, rEleIFNβ, hIFNα2a, and recombinant human interferon beta 1b (hIFNβ1b; Miltenyi Biotec., Bisley, UK). All dilutions were produced in DMEM medium and were made with equal masses of rEleIFNs and the corresponding human protein (IFNα: 500 ng/mL - 5 pg/mL; IFNβ: 1.67 µg/mL and 333.33 ng/mL - 30 pg/mL). Based on IU/ng data available for the hIFN preparations, these dilutions equated to 100,000-1 IU/ml. In two experimental replicates, EF cells were infected with BoHV-1 and at 0-, 8-, 12-, 16-, 24-, 48- and 72-hours post-infection (hpi), the EF medium was removed and replaced with 1 mL of the respective IFN solutions. Incubation in IFNs was continued for 24 hours before preparations were replaced once more with 1.5 mL EF medium. Plates were fixed, stained and assessed as described above.

## 3 Results

### 3.1 Identification of Asian elephant IFN genes and proteins

**Figure 1:**
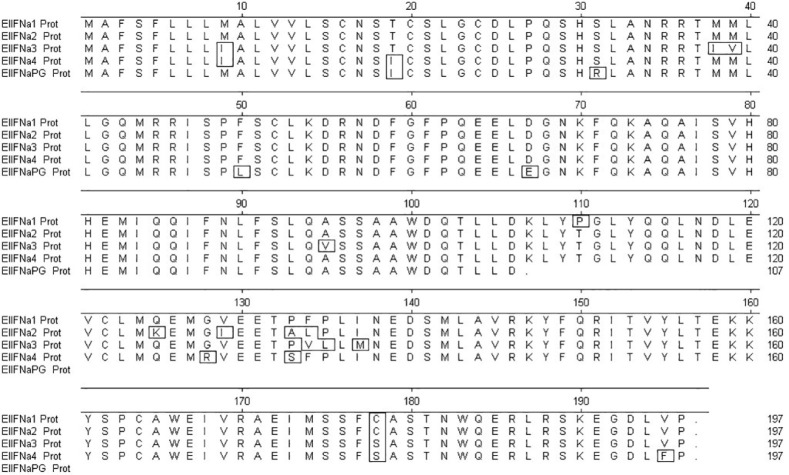
Multiple alignment of the amplified Asian elephant IFNα amino acid (AA) sequences using MegAlign software. The first 23 AA at the N-terminus of each protein are the predicted signal sequences. The stop codon within the nucleotide sequence of the IFNα pseudogene (IFNαPG) is at position 107. Boxed AAs are those that differ from the consensus.

Using predicted sequences from the African elephant and published sequences from the horse as templates, 13 distinct intronless IFNα-like sequences and a single IFNβ-like sequence were retrieved from the Asian elephant sequence assemblies available at the time. PCR-based amplification and sequencing identified four putatively functional Asian elephant IFNα genes, annotated in order of identification (EleIFNα1, IFNα2, IFNα3, IFNα4; GenBank accession numbers OR450024, OR450025, OR450026, OR450027). The EleIFNα1, IFNα2, IFNα3 and IFNα4 genes were each 591 base pairs in length and translated to proteins of 196 AA with predicted molecular weights of approximately 22.5 kDa. Upon alignment, the IFNα were 94.4-97.5% identical at the AA level. The fifth IFNα gene amplified was 600 base pairs in length, with a premature stop codon at position 107 of the AA sequence, demonstrating the identification of an Asian elephant IFNα pseudogene (GenBank accession number OR450028; EleIFNαPG; **Figure 1**). The 564-bp EleIFNβ gene identified encoded a putatively functional protein of 187 AA with predicted molecular weight of 21.6 kDa (GenBank accession number OR450029).

Using NCBI BLAST, the coding regions of the Asian elephant IFNα sequences had the highest homology with that of the African elephant with 91-99% and 86-98% identity at the NT and AA levels, respectively. Of similar immunogenetic relation were the Florida manatee (*Trichechus manatus latirostris*) sequences with 86-94% NT and 82-90% AA identity, whilst other mammalian species, including humans, were of significantly lower homology (53-78% identity at the AA level). Although the Asian and African elephant IFNβ AA sequences were 98.9% identical, the identity level dropped to 60% for human IFNβ (**Table 2**).

**Table 2:** Asian elephant IFN sequences obtained in this study and their percentage homology with selected mammalian species.

*E. maximus* gene (coding region only)	IFNα1	IFNα2	IFNα3	IFNα4	IFNαPG	IFNβ
GenBank accession number	OR450024	OR450025	OR450026	OR450027	OR450028	OR450029
Nucleotide (amino acid) sequence length	591 bp (196)	591 bp (196)	591 bp (196)	591 bp (196)	600 bp (106)	564 bp (187)
Predicted *L. africana*	91-99% (86-98%)					99.7% (98.9%)
Predicted *T. manatus*	86-94% (82-90%)					82% (68%)
*H. sapiens*	83-86% (71-77%)					77% (60%)
*E. caballus*	(74-78%)					(63%)
*S. scrofa*	(62-66%)					(68%)
*B. taurus*	(53-63%)					(59%)

Lengths of each nucleotide and amino acid sequence are shown (bp: base pairs). The range of percentage identities for the five Asian elephant IFNα genes are derived from a comparison to multiple nucleotide (and amino acid) sequences of the IFNα cluster of other species, available through the online NCBI BLAST tool.

### 3.2 Expression cloning of recombinant Asian elephant IFN

**Figure 2:**
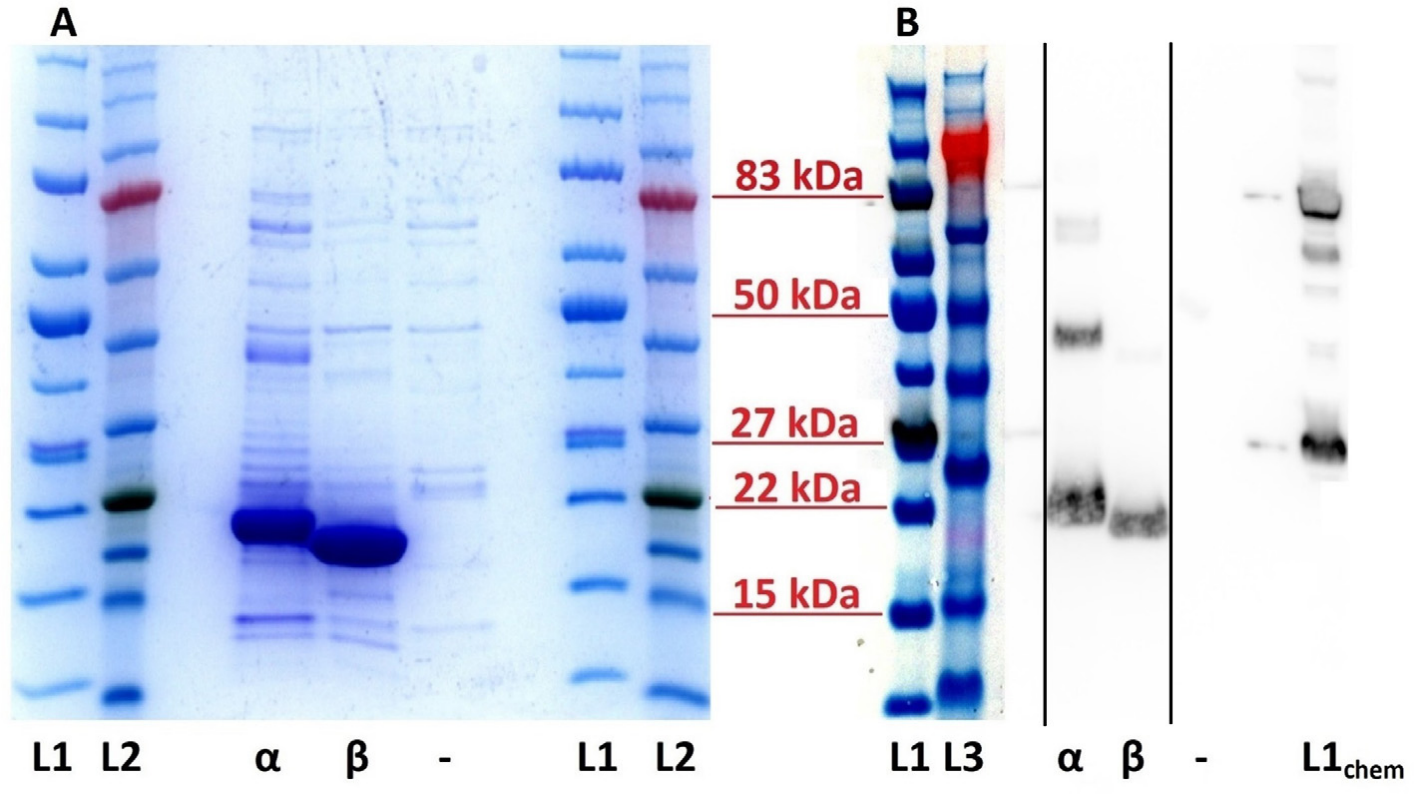
SDS-PAGE **(A)** and Western blot **(B)** of purified recombinant Asian elephant IFN proteins. **(A)** Bands of approximately 20 kDa and 19 kDa represent the rEleIFNα1 (α) and rEleIFNβ (β) proteins in the respective lanes. No significant corresponding protein expression could be seen in the control (–) preparations (pET303CT-His plasmid vector without IFN gene insert). **(B)** Using a monoclonal mouse anti-His tag antibody, positive chemiluminescent signal was observed for both rEleIFNα1 (α) and rEleIFNβ (β) at the expected respective sizes but not for control (–) preparations. Protein ladders are present on either side of each gel with relevant molecular weights indicated in red. L1: iBright™ Prestained Protein Ladder, L2: BLUeye™ Prestained Protein Ladder, L3: SeeBlue™ Pre-stained Protein Standard (all under visible light). L1chem: iBright™ Prestained Protein Ladder (under chemiluminescence). This figure is a combination of two separate gels, the original images of which have been submitted as **Supplementary Material**. Solid black lines in panel **(B)** indicate where sections of the original blot image have been spliced.

rEleIFNα1 and rEleIFNβ were successfully expressed, demonstrated by the presence of strong protein bands corresponding to the predicted sizes of 20.1 kDa and 19.2 kDa (**Figure 2**). Although co-purified contaminants were present, rEleIFNα1 and rEleIFNβ represented the substantial majority of proteins in each preparation, judged by the visible density of the corresponding protein bands in the Coomassie stained gels. Western blot analysis similarly confirmed expression of His-tagged rEleIFNα1 and rEleIFNβ. Whilst only a single protein band was visualised for rEleIFNβ, additional bands were observed in the rEleIFNα1 preparations, but at approximately double and triple the predicted molecular weight of the monomeric protein, possibly representing oligomerization. No chemiluminescent signal was recorded for control preparations.

### 3.3 Bovine herpesvirus infection model of EF cells

**Figure 3:**
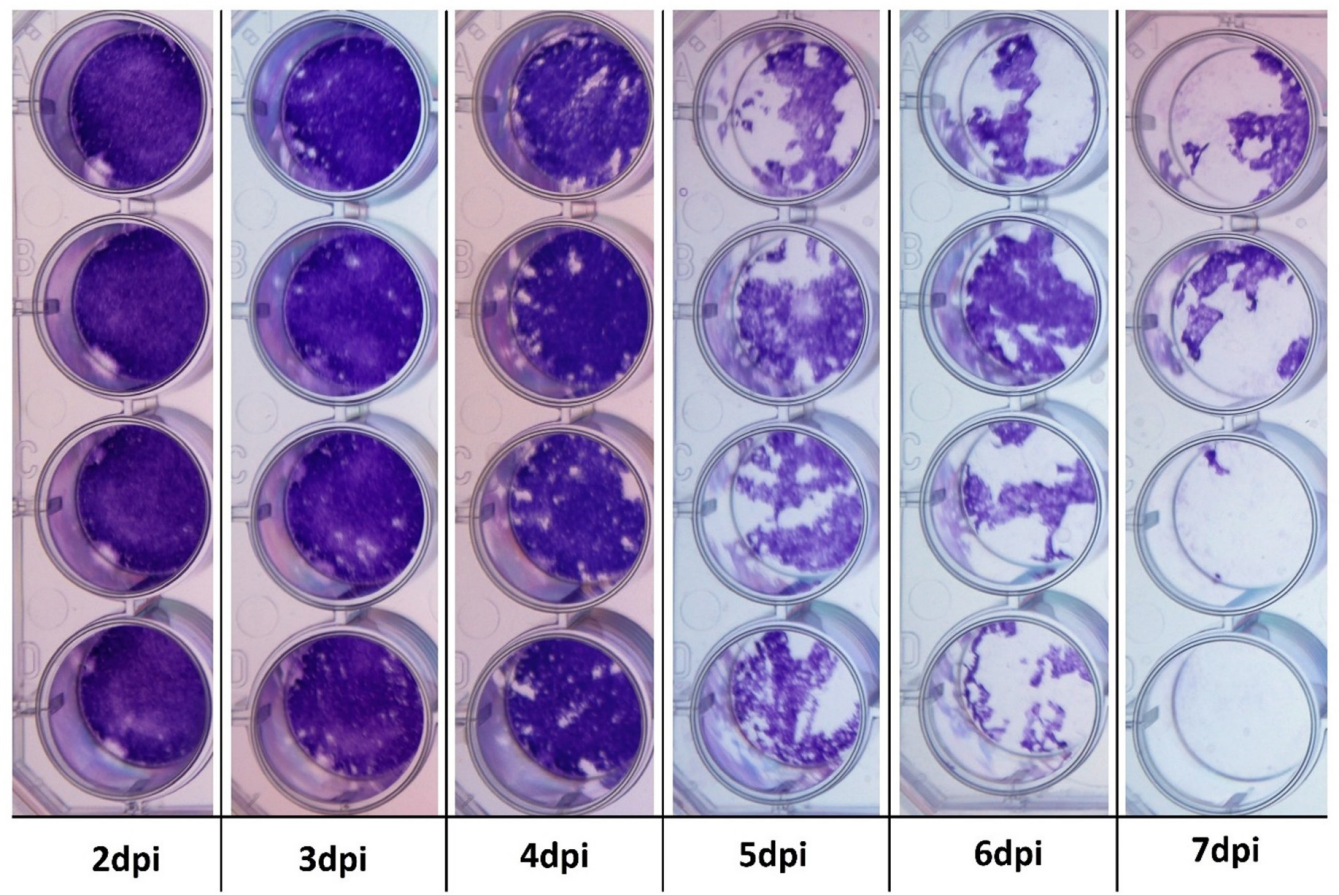
Cytopathogenic effect in BoHV-1-infected primary Asian elephant fibroblasts. Quadruplicate wells were infected with BoHV-1 and subsequently fixed and stained to evaluate for any cytopathogenic effects from 2 to 7 dpi. Viral plaques are evident at 3 dpi and monolayer integrity is lost beyond 5 dpi.

A surrogate system was required to investigate the antiviral activity of recombinant IFNs as no *in-vitro* EEHV infection model has yet been established (57–59). To do so, monolayers of EF cells were inoculated with either BoHV-1 or EHV-1 and the cells were fixed and stained 7 dpi to visualise CPE. Monolayers of EF cells were consistently and effectively destroyed by a 10^-3^ dilution of BoHV-1, but no CPE was observed in cells inoculated with a 10^-1^ dilution of EHV-1 (data not shown). To determine the kinetics of BoHV-1-in EF cells, quadruplicate wells were fixed and stained at 24-hour intervals from 2 to 7 dpi (**Figure 3**). Viral plaques were consistently visible at 3 dpi and destruction of the EF cell monolayer was evident within five days. Accordingly, a 10^-3^ dilution of BoHV-1 was used in all subsequent experiments to determine the antiviral bioactivity of IFNs in this study.

### 3.4 Bioactivity of human and Asian elephant IFNs in EF cells

**Figure 4:**
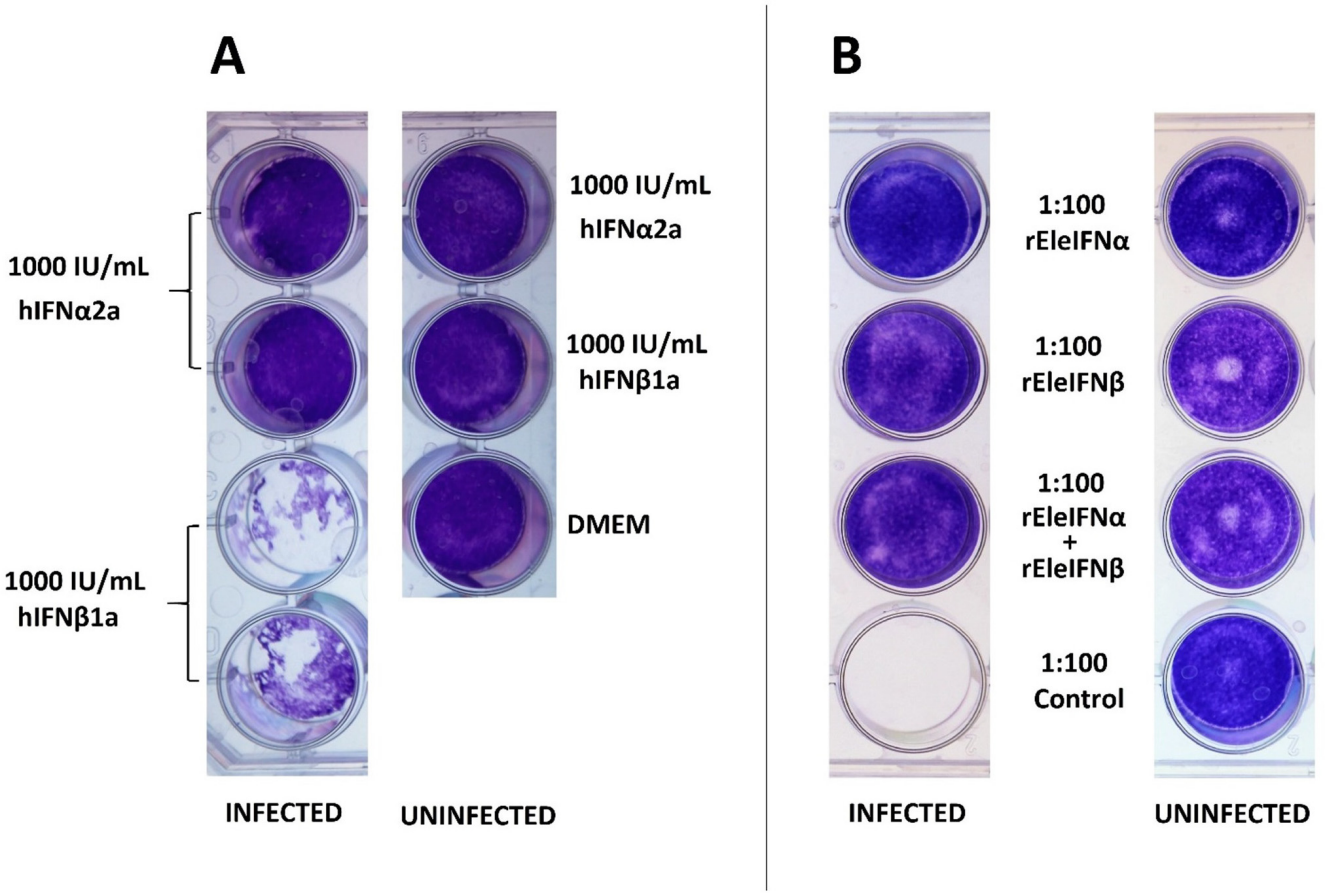
Anti-viral activity of recombinant human **(A)** and Asian elephant **(B)** IFNs in primary Asian elephant fibroblasts. EF cells were incubated in 1 mL dilutions of hIFNs or rEleIFNs for 8 hours prior to infection with BoHV-1 and cell monolayers were assessed 7 dpi. At the dilutions investigated, no protein preparations were cytotoxic in themselves and hIFNα2a, rEleIFNα1 and rEleIFNβ offered full protection from CPE. No protection was provided by hIFNβ1a or control preparations (protein preparations produced from pET303CT-His plasmid vectors without IFN gene insert).

Bioactivity of recombinant human and Asian elephant IFNs was initially demonstrated in an experiment using 1,000 IU/mL of rhIFNα2a or rhIFNβ1a or 1:100 dilutions of unquantified preparations of rEleIFNα1 or rEleIFNβ. EF cells were incubated with the IFNs for 8 hours prior to infection with BoHV-1. rEleIFNα1 and rEleIFNβ both protected cells from BoHV-1 induced CPE, whereas monolayers in control preparations were destroyed (**Figure 4**). Monolayers also remained intact following pre-exposure to 1,000 IU of hIFNα2a but were destroyed in wells pre-exposed to 1,000 IU of hIFNβ1a. No CPE was evident in uninfected cells. Considering the outcome of this experiment, the hIFNβ supplier was changed.

**Figure 5:**
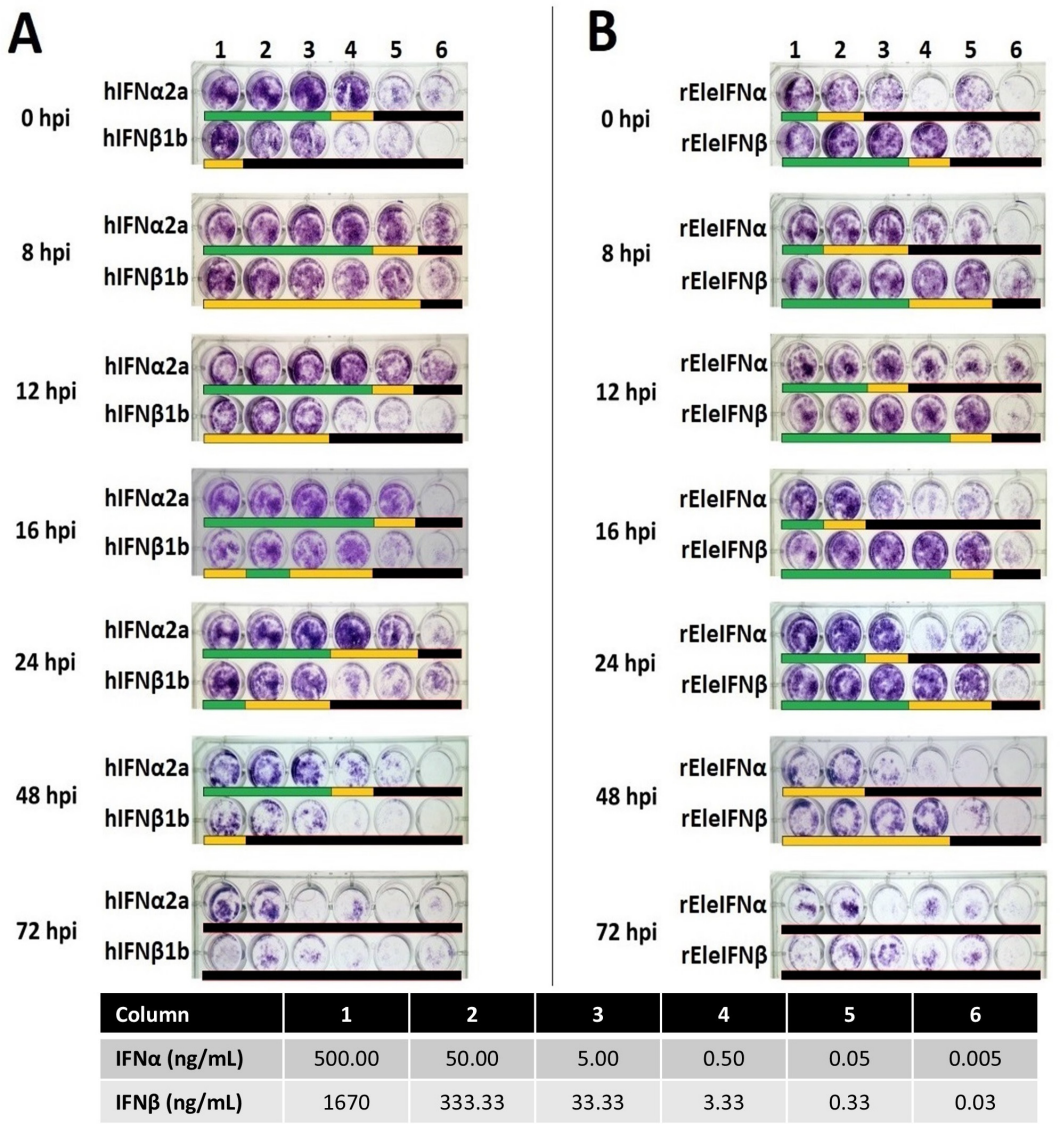
Kinetics of recombinant human **(A)** and Asian elephant **(B)** IFN bioactivities. EF cells infected with BoHV-1 were incubated in six dilutions of IFNs (Columns 1-6) for 24 hours at seven timepoints (0-72h) post-infection. Cell monolayers were assessed microscopically at 7 dpi. Beneath each well, a lack of CPE is denoted by a green bar, occasional (<5) viral plaques by a yellow bar and monolayer destruction by a black bar. Antiviral activity was observed with the application of hIFNα up to 48 hpi, with a minimum effective dose of 5 ng providing full protection from BoHV-1 infection. Up to 24 hpi, 33.3 ng of rEleIFNβ demonstrated potent antiviral activity. No antiviral activity was recorded for any IFN when applied beyond 48 hpi. Poor growth of EF cells was differentiated from CPE upon microscopic examination. Infected control wells (n=1 on every plate; not shown) demonstrated significant changes in morphology prior to death (e.g. shrinkage and clumping) whereas all uninfected control wells (n=1 on every plate; not shown) contained a population of healthy fibroblasts.

IFNs require a certain timeframe to exert their antiviral effects, and for rapidly growing viruses, pre-incubation of cells with IFN is essential for anti-viral protection. A further experiment was conducted to ascertain the timepoint at which IFN no longer protects EF cell monolayers and to compare relative bioactivities of recombinant human and Asian elephant IFNs. To do so, serial dilutions of IFNα (500 ng/mL - 5 pg/mL) and IFNβ (1.67 µg/mL and 333.33 ng/mL - 30 pg/mL) were applied at 0, 8, 12, 16, 24, 48 and 72 hpi. Based on data available for the rhIFN preparations, the dilutions tested ranged between 100,000 IU to 1 IU of IFN. Pre-exposure to at least 1,000 IU (5 ng) of hIFNα2a provided EF cells with full protection from BoHV-1 infection when administered up to 48 hpi (**Figure 5**). In contrast, 500 ng of rEleIFNα1 was required to consistently prevent CPE and protection was achievable only when applied up to 24 hpi. Cell monolayers were protected from BoHV-1 infection with only 33.3 ng of rEleIFNβ but the protective effect was also largely lost if applied beyond 24 hpi. No significant protective effect was observed using hIFNβ1b, indicating a difference in cross-species reactivity between human IFN alpha and beta. No protection from BoHV-1 infection was observed when IFNs were applied beyond 48 hpi.

## 4 Discussion

Here we identified five Asian elephant IFNα genes alongside a single IFNβ gene. Unsurprisingly, the Asian elephant gene sequences had the highest identity to those predicted from their closest relatives, the African elephant (*Loxodonta africana*) and Florida manatee (*Trichechus manatus latirostris*). Sequence diversity within the Asian elephant IFNα genes resembled those reported previously for other species, including humans (60–63). The repertoire of IFNα subtypes is most likely attributed to gene duplication events that have occurred throughout the evolution of vertebrate species, highlighting the vital role of these cytokines for their survival (1, 64). Gene duplication events may mitigate against replication errors but can also lead to the creation of pseudogenes, as identified here, and previously in both human and mouse IFN loci (11, 61, 65, 66).

Expression of recombinant proteins employed in this study was similar to previously published protocols (67–72). However, the lengthy dialysis in ever-decreasing concentrations of urea and guanidine hydrochloride was replaced by solubilisation of inclusion bodies with sarkosyl, Triton X-100 and CHAPS, as a more efficient method of recovering recombinant proteins (55, 56, 73). In the current study, a final concentration of 10% sarkosyl was necessary to obtain a sufficient amount of both His-tagged rEleIFNs that could be purified directly from the resulting solutions. Interestingly, both SDS-PAGE and Western blot analyses suggested the presence of homodimers of rEleIFNα1 and to a lesser extent, rEleIFNβ. Such a phenomenon has been documented for human IFNs, with as yet unknown biological consequences (74–77).

Subtypes of IFNα are known to possess distinct biological potencies (78–80), but here only EleIFNα1 was chosen for expression and purification as a proof-of-concept endeavour. As binding affinities of all subtypes of the human IFNα family have been demonstrated to be relatively comparable (81, 82), the expression of further rEleIFNα subtypes was not deemed a priority.

As IFNα family proteins are known to cross-react between animal species (83–86), it was considered prudent to compare bioactivity of the rEleIFNs to human IFN proteins in EF cells. Additionally, given the impact of EEHVs in this species, it was decided that an infection model using a herpesvirus was most pertinent to evaluate the antiviral bioactivity of the IFNs, rather than using the standard Vesicular Stomatitis Virus (VSV) model (87–89). The selection of BoHV-1 as a surrogate for EEHV was primarily due to the ongoing inability to culture the latter *in-vitro* (57–59). BoHV-1 and EHV-1 are known to induce a lytic infection in a variety of cell lines from different species (90–95). However, only BoHV-1 demonstrated a visible CPE in EF cells. Given that earlier studies have demonstrated a seroconversion of Asian elephants against BoHV-1 (96, 97) this result may not be as surprising as it initially appears. However, further work is needed to explore the lack of replication observed in relation to EHV-1.

Both recombinant Asian elephant IFNα and IFNβ proteins demonstrated significant antiviral activity against BoHV-1 infection of EF cells, when applied eight hours prior, and up to 24 hours post infection. The latter is perhaps not overly surprising, given that the development of a CPE was evident in untreated EF cells only after 72 hours.

As human IFNs are known to exert immune effects beyond the native species (50, 98–101), the bioactivity of human IFNα2a on EF cells was anticipated. However, the observation that human IFNβ had minimal (hIFNβ1b) or no (hIFNβ1a) bioactivity was surprising. This may be explained by the relatively distinct AA sequences of human IFNα and IFNβ, despite exerting their effects via the same receptor. Additionally, human and Asian elephant IFNα genes shared 71-77% sequence identity, whilst sequence identity for IFNβ gene was 60% (**Table 2**).

Interestingly, the protective effect of IFNs continued for the entire seven-day BoHV-1 study period, even after removal of IFNs, suggesting that IFN promoted a “head start immunity”, as proposed recently (102). Therefore, interferon signalling and ISG induction had occurred by, or continued beyond, the point of infection. The longevity of this effect is likely to be explained by the ongoing presence of the original virus inoculum and virus progeny, providing sustained interaction between the pathogen recognition receptors (PRRs) within the EF cells and the viral pathogen-associated molecular patterns (PAMPs). Indeed, recent work has demonstrated the expression of multiple anti-viral mediator genes, including IFNs, in Asian elephant white blood cells in response to the *in-vitro* administration of authorised PAMP-based veterinary medicinal products (103). A similar innate immune response, and the expression of innate immune mediators, is expected following exposure of EF cells to BoHV-1, but more crucially, following the exogenous administration of IFNs.

Recombinant IFNs displayed a positive correlation between the administered dose and the level of protection against BoHV-1. These preliminary dose-dependent responses were largely unaffected whether the IFNs were applied at the point of infection (0 hpi) or over the following 24 hours (8-24 hpi). The observed lack of protection at 48 hpi indicates that IFN signalling pathways need to have activated ISGs and anti-viral mediators before this point, whereafter certain viral proteins are expected to drive the immune-evasion of the IFN system (7, 104–107).

Since this study was conducted, a draft Asian elephant genome has been published (GenBank Bioproject PRJNA861314; assembly GCA_024166365.1, July 2022). However, although this assembly is not yet complete, it does contain some noteworthy information in relation to this study. Whilst the IFN locus cannot be described at this stage, all predicted IFN-I genes are located in chromosome 9 (of 27 autosomes). Of the 11 predicted IFNα genes, two are annotated as pseudogenes, with one having the pre-mature stop codon described here. Of the nine predicted functional IFNα genes, eight are annotated as IFNalpha-5-like, and one (XP_049750889.1) as IFNalpha-6-like. Importantly, the latter sequence has low identity score (<20%) and is shorter (182 AA) than all other predicted IFNα proteins, questioning the accuracy of its annotation. Whilst our annotation of IFNα1-4 is based on the chronological order of discovery, it is meaningful to highlight the fact that they are considered to represent four unique bioactive IFNα proteins. Notably, only the IFNα3, IFNαPG and IFNβ sequences described here are 99.5-100% identical to the draft genome, with a single mutation in IFNα3 likely representing a single nucleotide polymorphism. However, IFNα1, IFNα2 and IFNα4 are only 95-96% identical to the nearest match from the draft genome. It is unlikely that such differences are due to inter-animal variability and instead these IFNα sequences may represent duplications of IFNα not yet annotated in the draft genome, demonstrating the challenges associated with annotating genome areas with gene duplications. Additionally, since the current number of identified IFNα genes (eight or nine functional genes) is considerably lower than those in mammalian livestock species, humans, or mice, it is reasonable to assume that the IFNα1, IFNα2 and IFNα4 sequences identified in this study may represent additional genes to be added to the Asian elephant genome annotation.

Regarding the relative bioactivity of the two rEleIFNs, rEleIFNβ consistently provided the same level of protection as 15 times the same mass of rEleIFNα1. Although based on the previously mentioned assumption of an equal concentration of bioactive protein across the two preparations, this suggests that a similar hierarchy of affinity for the type I IFN receptor exists in Asian elephants as seen in humans and mice, where IFNβ demonstrates approximately 20 times greater affinity for the type I IFN receptor than IFNα (78, 79, 82). This would appear to make rEleIFNβ a preferred choice for clinical application. However, the preliminary demonstration of bioactivity of recombinant human IFNα in Asian elephant fibroblasts is an important consideration in relation to possible *in-vivo* applications, since pharmaceutical grade products, authorised for use in humans, are already available for veterinary prescription and administration.

The successful cloning and characterisation of rEleIFNs in this study, in combination with the development of a herpesviral infection model, provides an important preliminary means of demonstrating antiviral activity in Asian elephants. Work adding resolution to the Asian elephant genome (108) will aid in the description of the IFN-I cluster genome locus. Future work can accordingly document the remaining IFN genes, providing the basis to study their respective functions further, as documented in humans (78–80).

This study was designed as a preliminary investigation into whether the clinical application of rIFNs could contribute to the control of EEHV-HD, thereby maintaining the welfare of the captive population of Asian elephants worldwide, and aiding efforts for their conservation. In a recent case report, the clinical application of rhIFNα, along with a DNA immunostimulant, were considered to significantly contribute to controlling EEHV1A-HD in a juvenile Asian elephant, despite an exponential rise in viraemia (38). Importantly, the calf survived with no adverse effects. The same treatment protocol, but excluding IFNs or veterinary immunostimulants, had proven unsuccessful in a separate case of EEHV1A-HD at the same zoological collection less than 6 months earlier, highlighting the potential contribution of such biological products to disease control.

## Data Availability

The datasets presented in this study can be found in online repositories. The names of the repository/repositories and accession number(s) can be found in the article/**Supplementary Material**.

## Supplementary material

Supplementary Figure 1 A full scan of the entire original SDS-PAGE gel, shown in **Figure 2A**, of purified recombinant Asian elephant IFN proteins. Bands of approximately 20 kDa and 19 kDa represent the rEleIFNα (lane 4) and rEleIFNβ (lane 5) proteins in the respective lanes. No significant corresponding protein expression could be seen in the control preparations (pET303CT-His plasmid vector without IFN gene insert; lane 6). Protein ladders are present on either side of the gel: iBright™ Prestained Protein Ladder (lanes 1 and 8), BLUeye™ Prestained Protein Ladder (lanes 2 and 9).

Supplementary Figure 2 An uncropped and unedited visible light image of the entire original Western blot gel shown in **Figure 2B**. Protein ladders are present on either side of the gel: iBright™ Prestained Protein Ladder (lanes 1 and 11), SeeBlue™ Pre-stained Protein Standard (lanes 2 and 12).

Supplementary Figure 3 An uncropped and unedited chemiluminescence image of the entire original Western blot gel shown in **Figure 2B**. Using a monoclonal mouse anti-His tag antibody, positive chemiluminescent signal was observed for both rEleIFNα (lanes 4 and 5) and rEleIFNβ (lanes 6 and 7) at the expected respective sizes but not for control preparations (lanes 8 and 9). Protein ladders are visible on either side of the gel: iBright™ Prestained Protein Ladder (lanes 1 and 11).
