# Supplementary figures and images for: Asian elephant interferons alpha and beta and their anti-herpes viral activity
Source: Front Immunol. 2025 Mar 25;16:1533038. doi: 10.3389/fimmu.2025.1533038 (PMC11975597; doi:10.3389/fimmu.2025.1533038)

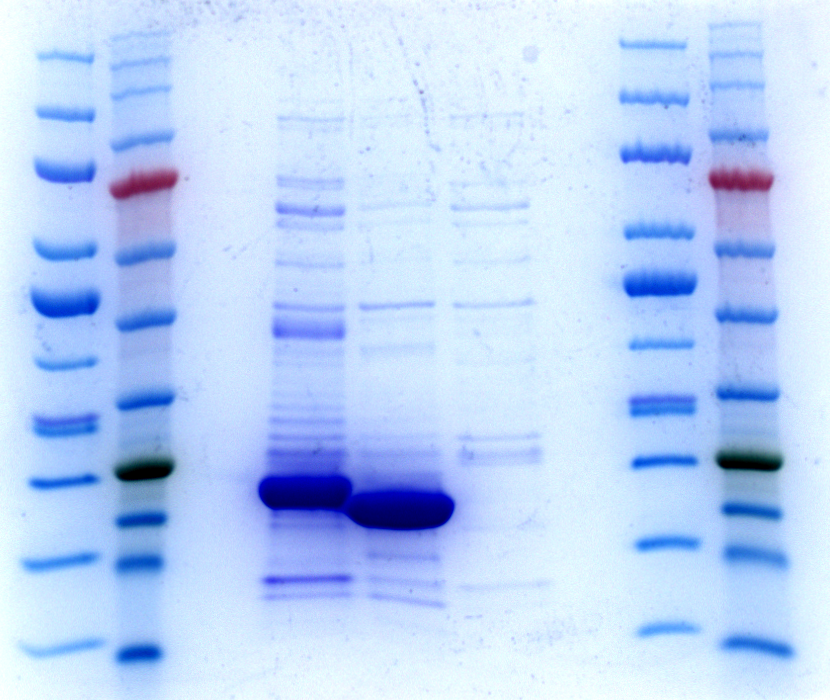

Supplement: Supplementary Figure 1 — A full scan of the entire original SDS-PAGE gel, shown in Figure 2A , of purified recombinant Asian elephant IFN proteins. Bands of approximately 20 kDa and 19 kDa represent the rEleIFNα (lane 4) and rEleIFNβ (lane 5) proteins in the respective lanes. No significant corresponding protein expression could be seen in the control preparations (pET303CT-His plasmid vector without IFN gene insert; lane 6). Protein ladders are present on either side of the gel: iBright™ Prestained Protein Ladder (lanes 1 and 8), BLUeye™ Prestained Protein Ladder (lanes 2 and 9). [file Image1.tif]

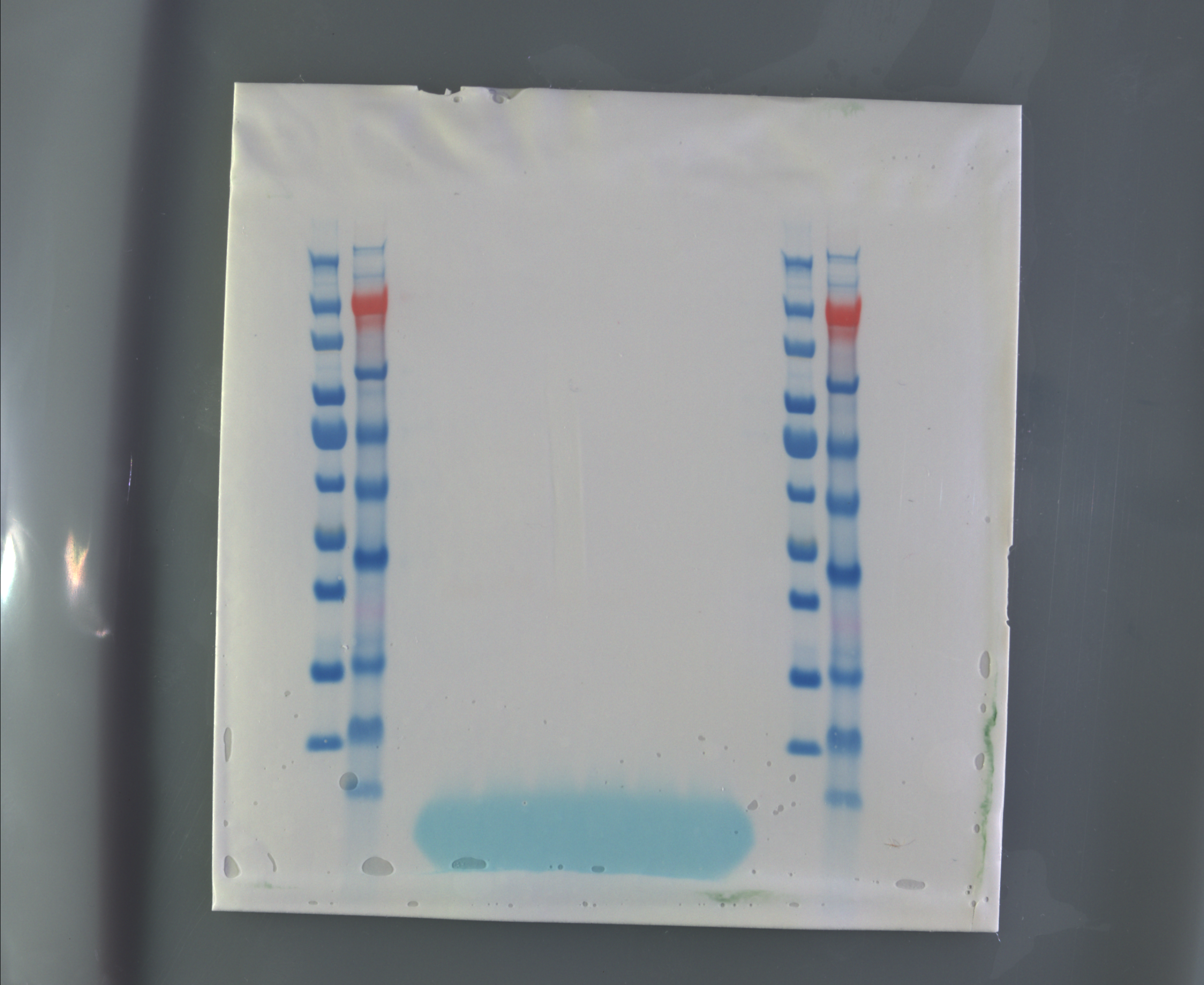

Supplement: Supplementary Figure 2 — An uncropped and unedited visible light image of the entire original Western blot gel shown in Figure 2B . Protein ladders are present on either side of the gel: iBright™ Prestained Protein Ladder (lanes 1 and 11), SeeBlue™ Pre-stained Protein Standard (lanes 2 and 12). [file Image2.tif]

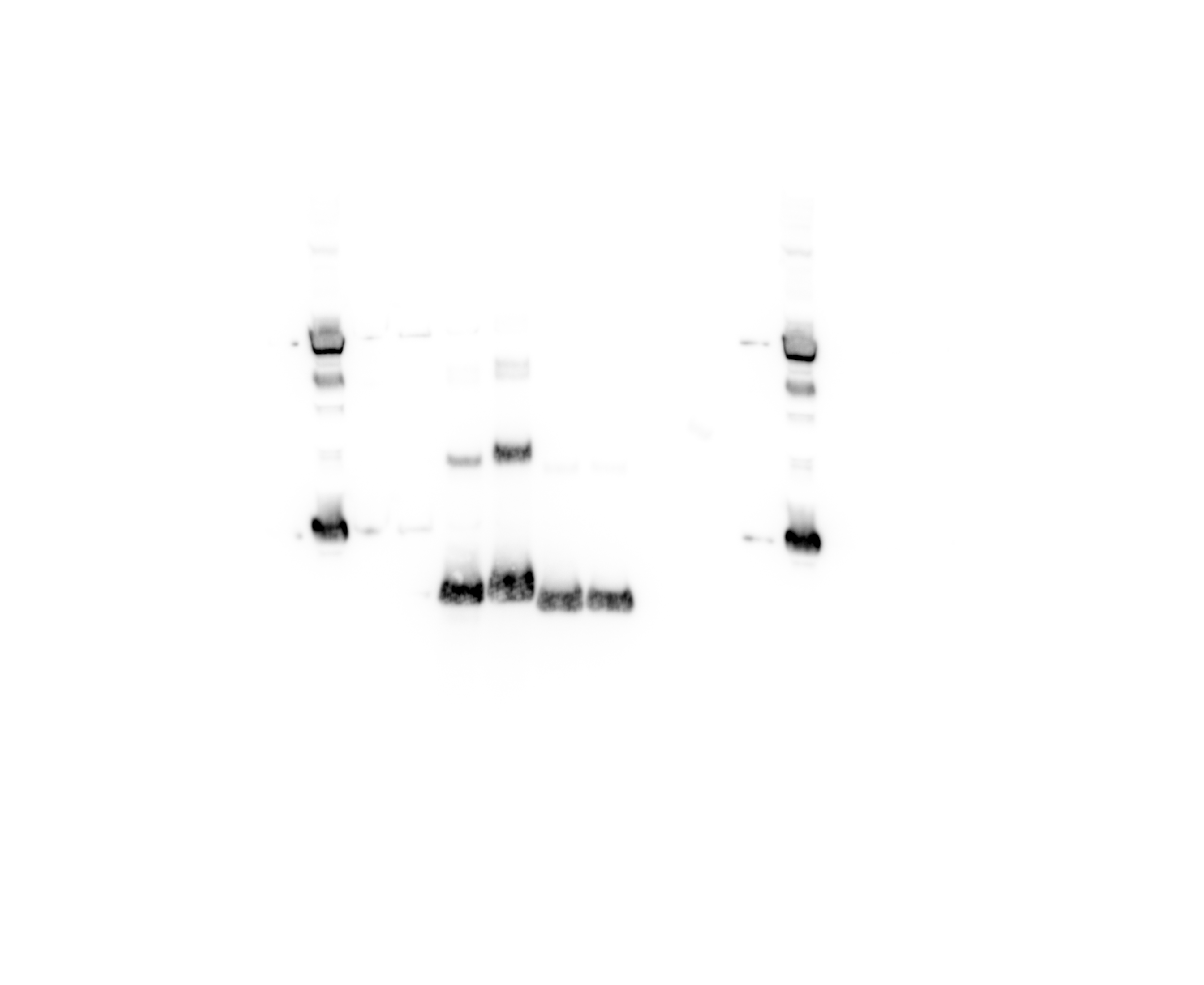

Supplement: Supplementary Figure 3 — An uncropped and unedited chemiluminescence image of the entire original Western blot gel shown in Figure 2B . Using a monoclonal mouse anti-His tag antibody, positive chemiluminescent signal was observed for both rEleIFNα (lanes 4 and 5) and rEleIFNβ (lanes 6 and 7) at the expected respective sizes but not for control preparations (lanes 8 and 9). Protein ladders are visible on either side of the gel: iBright™ Prestained Protein Ladder (lanes 1 and 11). [file Image3.tif]
